# Promising Antioxidant and Antimicrobial Potencies of Chemically-Profiled Extract from *Withania aristata* (Aiton) Pauquy against Clinically-Pathogenic Microbial Strains

**DOI:** 10.3390/molecules27113614

**Published:** 2022-06-04

**Authors:** Alhusain J. Alzahrani

**Affiliations:** 1 Almostakbal Medical Laboratories, Riyadh 36341, Saudi Arabia; ceo@almostaqbal-lab.com; 2 Department of Clinical Laboratory Sciences, College of Applied Medical Sciences, University of Hafr Al Batin, Hafar Al-Batin 31991, Saudi Arabia

**Keywords:** plants, bioactive, natural compounds, polyphenols, bacteria, free radicals

## Abstract

*Withania aristata* (Aiton) Pauquy, a medicinal plant endemic to North African Sahara, is widely employed in traditional herbal pharmacotherapy. In the present study, the chemical composition, antioxidant, antibacterial, and antifungal potencies of extract from the roots of *Withania aristata* (Aiton) Pauquy (RWA) against drug-resistant microbes were investigated. Briefly, RWA was obtained by maceration with hydro-ethanol and its compounds were identified by use of high-performance liquid chromatography (HPLC). The antioxidant activity of RWA was determined by use of ferric-reducing antioxidant power (FRAP), 1,1-diphenyl-2-picrylhydrazyl (DPPH), and total antioxidant capacity (TAC). The evaluation of the antimicrobial potential of RWA was performed against drug-resistant pathogenic microbial strains of clinical importance by use of the disc diffusion agar and microdilution assays. Seven compounds were identified in RWA according to HPLC analysis, including cichoric acid, caffeic acid, apigenin, epicatechin, luteolin, quercetin, and p-catechic acid. RWA had excellent antioxidant potency with calculated values of 14.0 ± 0.8 µg/mL (DPPH), 0.37 ± 0.08 mg/mL (FRAP), 760 ± 10 mg AAE/g (TAC), and 81.4% (β-carotene). RWA demonstrated good antibacterial potential against both Gram-negative and Gram-positive bacteria, with inhibition zone diameters ranging from 15.24 ± 1.31 to 19.51 ± 0.74 mm, while all antibiotics used as drug references were infective, except for Oxacillin against *S. aureus*. Results of the minimum inhibitory concentration (MIC) assay against bacteria showed that RWA had MIC values ranging from 2.13 to 4.83 mg/mL compared to drug references, which had values ranging from 0.031 ± 0.003 to 0.064 ± 0.009 mg/mL. Similarly, respectable antifungal potency was recorded against the fungal strains with inhibition zone diameters ranging from 25.65 ± 1.14 to 29.00 ± 1.51 mm compared to Fluconazole, used as a drug reference, which had values ranging from 31.69 ± 1.92 to 37.74 ± 1.34 mg/mL. Results of MIC assays against fungi showed that RWA had MIC values ranging from 2.84 ± 0.61 to 5.71 ± 0.54 mg/mL compared to drug references, which had values ranging from 2.52 ± 0.03 to 3.21 ± 0.04 mg/mL. According to these outcomes, RWA is considered a promising source of chemical compounds with potent biological properties that can be beneficial as natural antioxidants and formulate a valuable weapon in the fight against a broad spectrum of pathogenic microbes.

## 1. Introduction

Medicinal and aromatic plants are a natural reservoir of biologically active chemicals with therapeutic purposes that have been discovered and used by various groups of people for treatment throughout history [1]. Herbal medicine has remained the most economical and easily available form of therapy in many low-income countries [2]. Herbal medicines are currently used by almost 80% of people based in developing nations, which can be explained by people experiencing issues in obtaining modern medicines, or because alternative remedies are more acceptable from a spiritual and cultural standpoint [3]. A wide range of plants have been employed for medicinal reasons throughout history [4]. Numerous works have been conducted in recent years to investigate the diverse pharmacological effects of plant extracts, including antioxidant and antibacterial properties [5]. Plants have long been associated with improved human performance and health and are thus thought to be useful as part of diet therapy [6]. Many therapeutic compounds utilized in modern medicine were initially derived from medicinal plants; in fact, the process of generating new medications from herbal medicine is still ongoing, with significant breakthroughs occurring regularly [7]. Even though the theoretical notion of herbal medicine differs between nations throughout the world, the same plants are frequently used to treat the same or similar health problems worldwide [8].

Impacts of oxidative stress on human health have become a major concern. Our bodies produce more reactive oxygen species (ROS), including superoxide anion radicals, alpha-oxygen, hydrogen peroxide, singlet oxygen, and hydroxyl radicals, when we are stressed than enzymatic antioxidants, such as superoxide dismutase, catalase, and glutathione peroxidase, and non-enzymatic antioxidants (e.g., vitamin C), polyphenols, flavonoids, and carotenoids. This imbalance results in cell damage and serious health issues [9,10]. The development of degenerative diseases, such as cardiovascular diseases, osteoarthritis, malignancies, osteoporosis, neurological diseases, Alzheimer’s disease, Parkinson’s disease, and inflammatory diseases, is facilitated by a shortage of antioxidants, which can release reactive free radicals [11].

Antimicrobial resistance (AMR) arises when bacteria, parasites, viruses, and fungi can develop and proliferate in the presence of drugs designed to kill them [12]. One of the greatest threats to public health systems globally is the spread of AMR [2]. Patients are hospitalized longer, healthcare expenditures rise, and second-line drugs are more expensive because of AMR infections. Treatment failures are also a problem. In Europe alone, more than nine billion euros are spent each year to combat AMR [2]. At the same time, patients who are receiving treatment such as chemotherapy, dialysis, and surgery have challenges because of AMR, which weakens the human immune system’s capacity to fight against infections [13,14]. AMR will also have a significant impact on patients with chronic illnesses such as diabetes, cystic fibrosis, asthma, ulcerative colitis, chronic obstructive pulmonary disease, and rheumatoid arthritis [15]. Methicillin resistance in *Staphylococcus aureus* is one of the most known examples of AMR, which is related to significant mortality around the world [16,17]. Furthermore, drug-resistant bacteria have complicated the treatment of infections, particularly pneumonia and urinary tract infections [18].

*Withania aristata* (Aiton) Pauquy belongs to the genus *Withania* (family Solanaceae) that grows in North Africa and the Mediterranean basin [19]. It is traditionally used in the treatment of diseases including conjunctivitis, inflammation, stress, bronchitis, anxiety, ulcers, liver, and Parkinson’s disease [20]. The genus *Withania* includes multipurpose medicinal plants possessing numerous pharmacological properties, such as cytotoxicity against cancer cells, anti-inflammatory, analgesic, healing, immunomodulatory, anticholinesterase, and antioxidant properties [21,22,23,24,25,26,27,28]. In addition, plants of the genus of *Withania *have been reported to be effective for their adaptogenic, antibacterial, abortifacient, aphrodisiac, astringent, anti-inflammatory, deobstruent, diuretic, narcotic, sedative and tonic properties [29]. Recent works showed that species of the genus of *Withania* such as *W. somnifera* L. are multipurpose medicinal plants that are considered as a reservoir rich in pharmacologically active withanolides, which are a group of triterpenoids (i.e., steroidal lactones). These include withanolide A (g), withanoside IV (a), withaferin A (e), 27-hydroxywithanone (c), withanoside V (d), withastramonolide (f), withanone (h) withanolide B (i), and physagulin D (b) [30]. The biosynthetic pathways of secondary metabolites, such as withanolides, are tightly controlled by diverse temporal spatial cues, thereby limiting their concentrations in plant tissues. Moreover, the distribution of these secondary metabolites is mostly species-specific, where the distribution of a given plant species may be confined to a particular geographical location. The biosynthesis of phenolic compounds is largely controlled by several factors, including environmental conditions, nutritional status, and developmental stage, that ultimately affect plant growth and modulate transcriptional expression profiles of miRNA genes [31].

All plant parts of *Withania* sp., such as flowers, leaves, stems, roots, seeds, and bark, have medicinally important properties. This work was undertaken to investigate the chemical composition, antioxidant, antifungal, and antibacterial potencies of the ethanolic extract from the roots of *Withania aristata* (Aiton) Pauquy.

## 2. Results and Discussion

### 2.1. Chemical Composition of RWA

**Figure 1:**
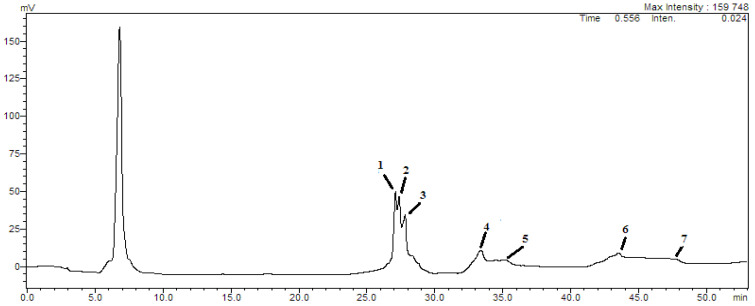
Liquid chromatographic HPLC analysis of RWA.

Analysis of the phytochemical composition of RWA by HPLC revealed interesting potentially active phenols, including cinnamic acid, caffeic acid, apigenin, epicatechin, luteolin, quercetin, and p-catechic acid (Figure 1; Table 1). Phytochemical identification by HPLC revealed that RWA was rich in polyphenols (622.17 mg/kg) and flovonols (765.23 mg/kg).

These findings are closely comparable to those found in previous works [32,33] reporting that catechin derivatives are likely to be the same major compounds found in tea. Similarly, p-coumaric acid, catechin, epigallocatechin-3-O-gallate, gallocatechin, and sinapic acid were also identified in different fractions of *Limoniastrum* [34]. Previous studies conducted on *Withania furtescens* revealed its richness in some phenolic compounds, including epicatechin, apigenin, caffeic acid, ferulic acid, and p-coumaric acid, which are almost similar to those found in RWA [26]. However, when compared to the chemical composition of crude extract from *Withania furtescens* (Table 2), the studied extract of *Withania aristata* (RWA) was somewhat different in terms of major compounds detected by HPLC (Table 1). In this context, *Withania aristata* was found to be higher in steroids such as withanosides, unlike the extract studied (RWA), which was found to be higher in polyphenols (Table 2) Gas chromatographic analysis (GC/MS) of extracts from the genus *Withania* showed many phenols, which is in agreement with the present study results [35,36].

The genus *Withania* is generally distinguished by the presence of withanolides, highly oxygenated C28 steroidal lactones which are identified from over 25 Solanaceae species, including Acnistus, Datura, Jaborosa, Nicandra and Mandragora [37,38,39]. Withanolides were also found in Taccaceae, Lamiaceae and Myrtaceae [40,41,42,43].

**Table 2:** Phytochemicals and pharmacological activities of the genus Withania.

Plant Name	Plant Part Used	Extract Type	Major Compounds	Pharmacological Activities	References
*Withania aristata*	Leaves	Crude extract	2-(4-hydroxy-3,5 dimethoxyphenyl)-3-oxetanamine and *N*-4-(3-furoylamine)-1-butanol	Anti-plasmodial activity	[44]
	Leaves	Isolation	Withanolides	cytotoxic activity	[45]
	Leaves	Isolation	Apocarotenoids, carotenoid, tetra-acetylated apocarotenoid, glucosides	Phytotoxic activity	[46]
	Leaves	Isolation	hydroxywithanolide F, withanolide A, withacoagulin	inhibitors of SARS-CoV-2 main protease (Mpro)	[47]
*Withania frutescens*	Leaves	Essential oil	camphor, thujone, carvacrol, and thymol	Antioxidant and antimicrobial activities	[20]
	Leaves	Essential oil	camphor, carvacrol, and thymol	Insecticidal and antifungal activities	[48]
	Leaves	Crude extract	Epicatechin, apigenin, caffeic acid, ferulic acid, and p-coumaric acid	Analgesic, anti-inflammatory, and healing activities	[26]
	Leaves	Crude extract	4β,17α,27-trihydroxy-1-oxo-22-R-witha-2,5,24-trienolide (1), 5β,6β-epoxy-4β,17α,27-trihydroxy-1-oxowitha-2,24-dienolide (2), and 2,3-dihydroxywithaferin A-3β-O-sulfate (3)	cytotoxic activity against cancer cell lines (HepG2 and HT29)	[49]
	Leaves	Total polyphenols	-	Antifungal and antioxidant activity	[50]
*Withania somnifera*	Roots	Crude extract	withanoside IV, physagulin D, 27-hydroxywithanone, withanoside V, withaferin A, withastramonolide, withanolide A, withanone, and withanolide B	Cytotoxic and pro-inflammatory enzyme inhibitory properties	[30]
	Aerial parts	Crude extract	isopelletierine, anaferine, withanolides, withaferins	Immunomodulatory activity, anti-inflammatory activities, anticancer and chemoprotective activities, hepatoprotective activity	[51]
	Leaves and roots	Crude extract	withaferin A and withanolide D	Antibacterial synergistic activities	[52]

### 2.2. Antioxidant Activity

**Figure 2:**
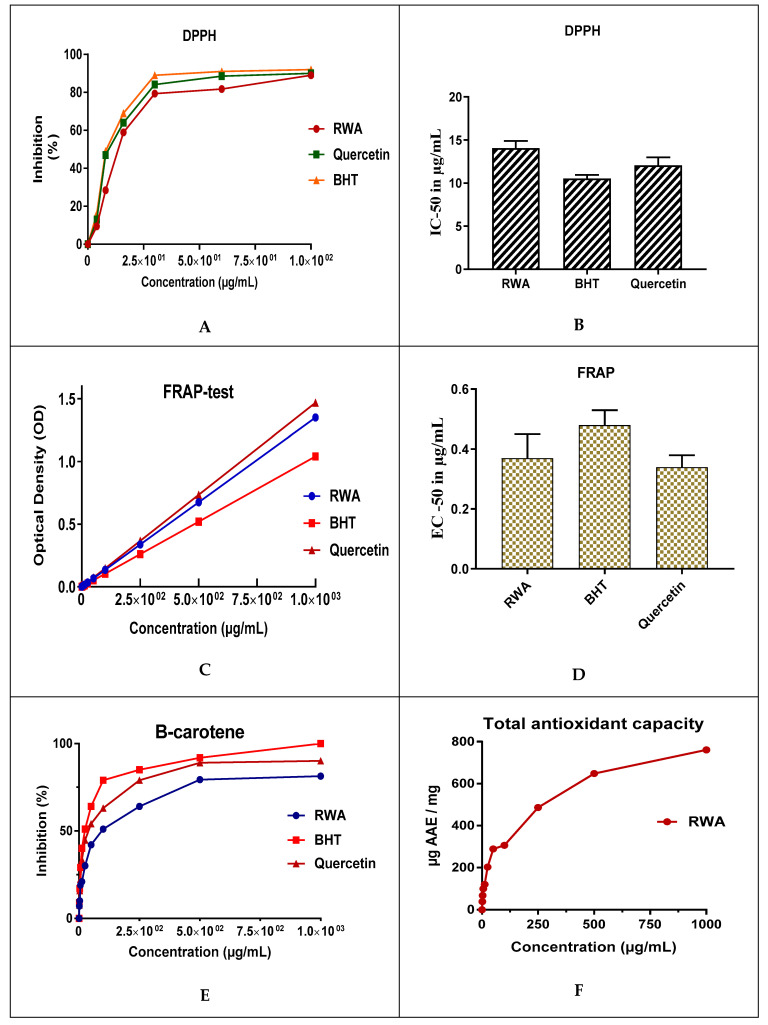
Antioxidant activity of RWA by DPPH (**A**,**B**), FRAP (**C**,**D**), TAC (**E**), and β-carotene (**F**).

The antioxidant activity of RWA was evaluated by four methods (DPPH, FRAP, TAC, and B-carotene). The antioxidant activity by DPPH revealed that the half-maximal inhibitory concentration (IC-_50_) of RWA was determined to be 14 ± 0.8 µg/mL, which is important when compared to BHT and quercetin which recorded 10.5 ± 0.4 and 12 ± 1 µg/mL (Figure 2), respectively. *Withania somnifera* root extract exhibited an IC-_50_ of 18 µg/mL, which is greater than that recorded for RWA. Notably, the root extract of *Withania frutescens* recorded an IC-_50_ value of 10 µg/mL [50]. The FRAP method revealed that the half-maximal effective concentration (EC-_50_) recorded for RWA was 37 ± 0.08 mg/mL, which is less (more potent) than that recorded for controls: 0.48 ± 0.05 (BHT) and 0.34 ± 0.04 (quercetin) [50].

To the best of our knowledge, no previous study has investigated the antioxidant power of *Withania aristata* hydro-ethanol extract (Table 2), and hence other species in the genus Withania were used for performing a comparison. From Table 2, it can be seen that *Withania frutescens* and *Withania somnifera* possessed antioxidant activities. Notably, the total antioxidant capacity of RWA was 760 ± 10 µg AAE/mg (Figure 2), which is higher than those recorded for root extracts (478 ± 38 µg AAE/mg) and leaf extracts (317 ± 47 µg AAE/mg) of *Withania frutescens* [40], and even higher than that recorded for the extract from *Withania somnifera* (45.41 ± 0.018 µg AAE/mg) [51]. Antioxidant activity of B-carotene revealed that the free radical scavenging activity of RWA was 81.4% ± 1.1%, and 90.1% ± 1.4% for quercetin (Figure 2), which is higher than those recorded for the root extract (57%) and leaf extract (36%) of *Withania frutescens* [50]. In addition, the anti-free radical activity of RWA was higher than those recorded for Smilax leaves’ extract (71.5% ± 0.91%) [52]. Notably, the remarkable difference in antioxidant powder of RWA and other species in the genus *Withania* could be due to the difference in their chemical composition, which shows that *Withania frutescens* extract is higher in camphor, thujone, carvacrol and thymol [53]. Moreover, *Withania somnifera* extract is higher in withanolide A, 12-deoxy withastramonolide and withaferin A [30]. In addition, edaphic and environmental factors affecting the plant growth could modify its chemical composition, and therefore its antioxidant power [54]. The anti-free radical activity of natural compounds is due to phenolic phytochemicals [55]. The presence of phytochemicals such as phenolics, flavonoids, alkaloids, saponins, steroids, glycosides, tannins, protein, and amino acids in plant extracts is responsible for their antioxidant properties [56,57].

The antioxidant power shown in the studied plant extract might be attributed to the presence of phenols identified by HPLC, such as cichoric acid, caffeic acid, apigenin, luteolin, quercetin, and catechin, which might work singly or in concert to scavenge free radicals [32]. In this aspect, luteolin-7-diglucuronide, which is recognized for its antioxidant potential, notably 1-O-caffeoyl glucose, demonstrated significant antioxidant activity with DPPH radical scavenging [58]. The antioxidant activity of *Petroselinum crispum* was most likely attributed to apigenin-O-pentoside [59]. Cichoric acid identified in the RWA possessed a powerful antioxidant effect by acting on iron-chelating pro-oxidant metals [60]. The antioxidant properties of medicinal plants are due to flavonoid and phenolic chemicals, which are plentiful in several vegetables, fruits, and flowers [61]. Plant-based antioxidant phytochemicals, especially total polyphenols and flavonoids, have been used in herbal medicine to treat various diseases, such as cancer, aging, diabetes, and cardiovascular diseases [62].

### 2.3. Antibacterial Activity

As shown in Table 3 RWA evinced potent antibacterial activity against all bacterial strains. RWA strongly abolished the development of Gram-positive and Gram-negative bacteria, resulting in relatively large zones of inhibition whose diameters ranged from 15.24 to 19.51 mm. These findings can be considered more important when compared to commercially available antibiotics, which were almost ineffective against the studied bacteria. Notably, all bacterial strains were drug-resistant, except for *E. coli* and *S. aureus*, which were slightly sensitive to Oxacillin, resulting in inhibition zones of 11.71 and 9.89 mm (Table 2), respectively. The MICs of RWA against all bacteria were in the range of 2.13 to 4.83 mg/mL, which was interesting when compared to those of commercially available antibiotics (Table 4).

From Table 3 and Table 4, it can be seen that RWA has excellent antimicrobial activity even at low concentrations against almost all tested strains. As far as we know, no previous study has investigated the antibacterial effect of *Withania aristata* hydro-ethanol extract (Table 2), and hence other species in the genus Withania were used for performing a comparison. From Table 2, it can be seen that extracts from *Withania frutescens* and *Withania somnifera* possessed antibacterial activity. Notably, our results are in good agreement with those reported elsewhere [63], wherein it was reported that *Withania* species, particularly *W. frutescens* extract, possessed better antibacterial activity against Gram-positive, *Staphylococcus aureus* and *Streptococcus pneumoniae,* and Gram-negative, *Escherichia coli* and *Klebsiella pneumoniae*, bacteria. Similarly, *W. frutescens* polyphenols extracted from the roots showed a moderate antibacterial effect against Gram-positive and Gram-negative bacteria, resulting in inhibition zone values up to 13.5 mm and MIC values up to 5 mg/mL [50]. A previous study on methanol root and leaf extracts along with the *n-*hexane leaf extract of *W. frutescens* at 1 mg/disc revealed that only the leaf methanol extract exhibited antibacterial activity [25]. Previous reports identified alkaloids in *W. somnifera* root extracts as potentially responsible phytoconstituents for antibacterial properties against *E. coli*, *Staphylococcus aureus*, and *Salmonella typhimurium* [64].

The antimicrobial effect presented here can be due to compounds detected in RWA by HPLC, such as cichoric acid, caffeic acid, apigenin, luteolin, quercetin and catechin (Table 1). The RWA studied in this work is composed of phenols that may act synergistically or separately to kill bacteria. The cinnamic acids were reported to have effects against *E. coli*, *S. aureus*, *Streptococcus* sp., *C. albicans,* and *A. niger*, with MIC values of about 5, 6.75, 0.84, 0.40, and 0.84 mM, respectively [65]. Notably, *B. cereus*, *B. subtilis*, *S. lutea*, *E. coli*, *P. aeruginosa*, and *S. typhi* were killed by extracts from *Luffa cylindrical* that were high in 1-O-caffeoyl glucose [57]. Luteolin could be one of the compounds responsible for the antibacterial effects of RWA, because it was found that this compound had powerful antibacterial effects against *S. aureus* [66]. *S. typhi*, *S. aureus,* and *Klebsiella pneumonia* were successfully inhibited by ethanol extract of *Petroselinum crispum,* that was found to be rich in apigenin [67]. Quercetin extracts from *Persicaria perfoliata* were also reported to be efficacious against *Escherichia coli*, *Propionibacterium acnes*, and *Staphylococcus aureus* [39]. Hydroxycinnamate polyphenols such as caffeine acid, p-coumaric acid, and ferulic acid can slow the growth of bacteria such as *E. coli* and *Staphylococcus aureus* [43]. Gram-positive *Bacillus cereus* and *Micrococcus luteus* were shown to be the most susceptible to caffeic acid in a different investigation, while *E. coli* and *Staphylococcus aureus* were the most resistant bacteria [68]. For the examined drug-resistant bacteria, the MIC value was 4 mg/mL, and the diameter of inhibition ranged from 10.52 to 19.12 mm with 10 µL of apigenin [69]. Polyphenols’ antimicrobial activity is influenced by the degree of hydroxylation and their structural characteristics, which make bacteria vulnerable to polyphenols [20].

A possible mechanism for the polyphenol-induced mortality in susceptible strains is their impact on the cytoplasmic membrane, which disrupts polysaccharides, fatty acids, and phospholipids [70].

### 2.4. Antifungal Activity

As shown in Table 4, RWA demonstrated interesting antifungal properties against all fungal strains used for the experiment. Notably, RWA recorded promising inhibition zone diameters against all fungal species, with calculated values ranging from 25.65 to 29 mm. Similarly, RWA recorded low MIC values (more potent) against all fungal species, with calculated values ranging from 2.04 to 5.71 mg/mL.

To our knowledge, no previous literature has described the antibacterial effect of *Withania aristata* hydro-ethanol extract (Table 5), and hence other species in the genus Withania were used for discussions. From Table 5, it can be seen that extracts of *Withania frutescens* exhibited an antifungal effect. Notably, RWA showed very promising results of antifungal activity against fungal species such as *A. niger*, *C. albicans*, *F. oxysporum,* and *A. flavus*, which are comparable with previous work [20], reporting that the extract from *W. adpressa* weakly inhibited the *Penicillium italicum* growth. Similarly, *W. frutescens* extract was active against *A. niger*, *C. albicans*, *F. oxysporum,* and *A. flavus* [48]. The antifungal effect of RWA is most probably due to chemicals detected in its phytocomponents, such as cinnamic acid, caffeic acid, apigenin, epicatechin, luteolin, quercetin, and p-catechic acid. Cinnamic acid (C_9_H_8_O_2_) is widely used as a food additive and as an antibacterial agent because of its ability to control *A. niger* [71]. Fungal strains, particularly *Candida albicans,* were shown to be the most sensitive to caffeic acid [68].

Microbial growth rates were reduced by cinnamic acid because it damaged their plasma membrane and caused oxidative stress in their cells [72]. The antifungal potency of natural products may result from direct damage to the membrane, leading to microorganism death. Polyphenols are most likely to function as a cell membrane lubricant. Thymol and p-cymene oil were shown to be effective against *Candida* species because of direct damage to the cell membrane [73]. The susceptibility of microbes to polyphenolic compounds can be explained by their effect on the microbial membrane owing to their high lipophilicity. This results in severe structural perturbations of its polysaccharide and lipidic fractions, thereby leading to disruption of membrane permeability and eventually microbial mortality [74].

## 3. Conclusions

Results of the current study revealed that ethanolic root extract from *Withania aristata* (Aiton) Pauquy exhibits potent antioxidant and antimicrobial properties against clinically isolated drug-resistant pathogens. In light of these findings, RWA might be used as an alternative to conventional antibiotic therapy. The mechanisms of action (MOA) of RWA are still unknown, however it is plausible that a complex combination of factors might exert diverse biological interactions simultaneously and synergistically. Consequently, future research will focus on identifying the MOA of individual pure compounds. Evaluation of the possible adverse effects on non-target species, as well as pre-clinical and clinical investigations in non-human primates and people, will be required before RWA may be used as a natural treatment to control germs.

## 4. Material and Methods

### 4.1. Growth Media and Chemical Material

Mueller–Hinton Agar (MHA) and Mueller–Hinton Broth (MHB) were used as growth media for bacteria, while Sabouraud Dextrose Agar (SDA) and Sabouraud Dextrose Broth (SDB) were used for antifungal activity. All growth media were purchased from Biokar, France. The control positive chemicals used in this work were Kanamycin (KAN), Oxacillin (OXA), Streptomycin (STR), and Ceftizoxime (CZX) as antibiotics, and Fluconazole (FLU), which were purchased from Sigma-Aldrich.

### 4.2. Plant Material

*Withania aristata* (Aiton) Pauquy was collected from Maghrebian Sahara in March 2021, the period for flowers. After being identified by a botanist, the plant was deposited at the University Herbarium under the reference A2/WDBF21. Consequently, the roots were cleaned and dried in the dark in a ventilated area for 8 days before extraction.

### 4.3. Preparation of Plant Extract

The dried roots were ground by use of an electric grinder in fine powder, which was extracted by mixing 10 grams with 70 mL of ethanol and 30 mL of distilled water. The whole solution was left for 24 h under agitation at room temperature. After filtration, the extract was concentrated under reduced pressure to obtain a dry extract, which was stored at 4 °C until further use.

### 4.4. HPLC/MS Analysis

Separation and identification of compounds in RWA were conducted by use of protocols standardized [26]. To achieve this goal, a Shimadzu HPLC/MS (Kyoto-Japan) equipped with a DGU-20A5R degasser, a CBM-20A controller, a CTO-20AC column oven, two LC-20AD dual-piston parallel flow pumps, and a SIL-30AC autosampler was used, via an electrospray (ESI) source, SPD-M20A photodiode array detector, and LCMS-20A mass spectrometer, CTO-20AC column oven, SIL-30AC autosampler, SPD-M20A photodiode array detector, and LCMS-2020 mass spectrometer (Shimadzu, Kyoto, Japan). The compounds were separated using a C18 column (150 mm/4.6 mm/2.7 m) with a mobile phase of water–acetic acid (99.80/0.20 *v*/*v*, solvent A) and acetonitrile–acetic acid (99.80/0.20 *v*/*v*, solvent B). Prior to ESI/MS detection, the flow rate was fixed at 1000 L/60 s and divided at 200 L/60 s. The following gradient elution program was used: 0–5 min, 5% B, 5–15 min, 10% B, 15–30 min, 20% B, 30–60 min, 50% B, and 60 min, 100% B, at 280 nm. Negative mode was employed for the mass spectra settings: scan range (m/z), drying gas flow rate (15 L/60 s), scan speed (2500 amu/s), nebulizing gas flow rate (1.5 L/60 s), event time (0.5 s), thermal block temperature (300 °C), and interface temperature (350 °C). The quantification of these compounds was based on the polyphenolic compound calibration curves: caffeic acid, cinnamic acid, rosmarinic acid, luteolin, apigenin and quercetin.

### 4.5. Antioxidant Activity

The antioxidant activity of RWA was determined by the use of three bioassays, including ferric-reducing antioxidant power (FRAP), 1,1-diphenyl-2-picrylhydrazyl (DPPH), and total antioxidant capacity (TAC). BHT (butylhydroxytoluene) and quercetin were used as positive controls.

#### 4.5.1. DPPH Test

The DPPH test was used according to the protocol described by Bourhia and co-authors [75]. In tubes, 750 µL of RWA at various concentrations (0.001 to 1 mg/mL) and 1500 µL of DPPH (2.4 mg/100 mL) were introduced; after shaking, the tubes were placed in the dark at room temperature for 30 min. The reading was taken by measuring the absorbance at 517 nm. The results were expressed as free radical inhibition in percentages (I %) by use of the following formula:

I (%) = [1−(Abs sample−Abs negative of control)] × 100]

where I (%) is the percentage of free radical scavenging activity (AAR%), Abs sample is the absorbance of the sample, and Abs negative control is the absorbance of the negative control.

#### 4.5.2. FRAP Test

Briefly, 0.2 mL of RWA with concentrations ranging from 0.001 to 1 mg/mL was combined with 3.8 mL of FRAP reagent. FRAP reagent was obtained by mixing 300 mM of sodium acetate at pH 3.6, 10 mM of TPZT (tripyridyltriazine), and 20 mM of FeCl_3_ hexahydrate (10:1:1, *v*/*v*). The mixture was incubated for 30 min at 36 °C, and the absorbance was measured at 593 nm. Quercetin and BHT served as positive controls in this experiment [76].

#### 4.5.3. Total Antioxidant Capacity

This test was performed by use of the phosphomolybdate method (PPM; 4 mM ammonium molybdate, H_2_SO_4_ (0.6 M), and Na_3_PO_4_ (28 mM)). In tubes, 200 µL of RWA at different concentrations (0.001 to 1 mg/mL) was shaken with 2000 µL of PPM. Next, tubes were incubated in a water bath (95 °C/90 min) before reading the absorbance at 695 nm. The negative control consisted of PPM and methanol [77].

#### 4.5.4. β-Carotene Bleaching Assay

Oxidation of linoleic acid generates peroxide radicals following the abstraction of hydrogen atoms from diallyl methylene groups of linoleic acid [4]. Briefly, 1 mg of β-carotene was solubilized in 2000 µL of chloroform and added to 50 µL of linoleic acid and 500 µL of Tween 40. Afterward, the mixture was evaporated to remove the solvent, and then 50 mL of hydrogen peroxide 30% (H_2_O_2_) was added to the dry residue to obtain an emulsion. A blank was prepared without β-carotene to calibrate the spectrophotometer. Next, 100 µL of RWA was added to 2.5 mL of the prepared emulsion, after incubation at 50 °C for 2 h. Free radicals oxidized the unsaturated β-carotene, resulting in decreasing the absorbance [5]. The percentage of inhibition was determined as follows: 

Antiradical activity (%) = (RWA absorbance/BHT absorbance) × 100

### 4.6. Antimicrobial Activities

The antimicrobial effect was evaluated against nine microbial species, including five multi-resistant bacterial strains involved in nosocomial infections: *Escherichia coli*, *Klebsiella pneumoniae*, and *Acinetobacter baumanii* as Gram-negative bacteria, and *Staphylococcus aureus* and *Streptococcus pneumonia* as Gram-positive bacteria. In addition, one clinical yeast strain, namely *Candida albicans*, and three strains of mold, including *Aspergillus niger*, *Aspergillus flavus,* and *Fusarium oxysporum,* were also used for testing.

#### 4.6.1. Microbial Inoculum Preparation

The bacterial and the yeast suspensions were prepared by the same method. Briefly, two microbial colonies were collected from a fresh culture after 24 h in Mueller–Hinton agar (MHA) and Sabouraud Agar (SBA) media and then suspended in 0.9% NaCl solution. Subsequently, the suspension turbidity was adjusted to be 0.5 McFarland [78]. For the mold species, the inoculum was prepared by the conidial suspension. Briefly, sporulation was obtained by culturing the mold strains on SBA at 27 °C for 5 days. Next, the conidia were collected by flooding the plate with 5 mL of 0.05% (*v*/*v*) Tween 20 using a sterile spreader. The number of conidia in the solution was counted by use of a hemocytometer (area: 0.0025 mm^2^, depth: 0.2 mm) with optical microscopy before being adjusted to be 10^6^ conidia/mL in 0.9% NaCl solution [79].

#### 4.6.2. Disc Diffusion Method

The antimicrobial potential was assessed by use of the disc diffusion bioassay [80,81]. Briefly, 9 cm Petri dishes containing MHA and SBA were inoculated with 1 mL of fresh and adjusted microbial inoculum, and then the Petri dishes were dried for 10 min at ambient temperature. Next, discs measuring 6 mm in diameter were impregnated with 5 μL of RWA (1 mg/L), and positive controls (50 μg Kanamycin/mL, 50 μg Oxacillin/mL, 50 μg Streptomycin/mL, 50 μg Ceftizoxime/mL, and 50 μg Fluconazole/mL) previously dissolved in 5% DMSO were placed on the medium surface and left for 4 h at 4 °C to allow compound diffusion. Consequently, dishes were incubated at 37 °C for the bacteria and 30 °C for the yeast for 24 h, while molds were incubated for 7 days at 27 °C. After incubation, the antimicrobial activity was expressed as the mean of inhibition diameters (mm).

#### 4.6.3. Determination of Minimum Inhibitory Concentration (MIC)

The MICs were determined by microdilution assays in 96-well microplates [63]. Briefly, the RWA was diluted in agar (0.2%), whilst the positive control was suspended in MHB and SBB media in sterile hemolysis tubes supplemented with 5% DMSO. Following that, 50 μL of each medium was deposited in each well of the microplate. Afterward, 100 μL was used as a matter solution to prepare a range of concentrations using dilution-based factor 2. Inoculation was carried out by depositing 50 μL of the microbial suspension in all wells, except the first well which served as a negative control for growth. Finally, the microplate was incubated for 24 h at 37 °C for the bacteria strains and 30 °C for the yeast strain. After incubation, 20 μL of (1%) 2,3,5-triphenyl tetrazolium chloride (TTC) was added to all wells prior to reading the absorbance. Wells containing bacterial growth became pink due to the activity of the dehydrogenases, while the wells without bacterial growth remained colorless after 2 h of incubation. Therefore, MIC corresponds to the lowest concentration, whereby no pink color appeared. To evaluate the antifungal activity (mold strains), the macro-dilution method was used [79]. First, all the tested concentrations were diluted in a PDB (potato-dextrose agar) medium. The final concentration in each tube was calculated for a final volume of 5 mL. Thereafter, all tubes were inoculated with 100 μL of the fresh fungal conidia previously adjusted to be 10^6^ conidia/mL. Finally, tubes were incubated in a shaker to mix the contents for 5 days at 27 °C. After incubation, the MIC was the lowest concentration of antimicrobial agent that completely inhibits the growth of microbes in tubes.

### 4.7. Statistical Analysis

Statistical analysis was conducted by use of GraphPad Prism version 7.0 software. Normality was verified by use of the Shapiro–Wilks test, and homogeneity by the *t*-test. Analysis of variance was performed, with Tukey’s HSD test as a post hoc test for multiple comparisons. A significant difference was considered at *p* < 0.05.

## Figures and Tables

**Table 1:** Chemical compounds identified by HPLC analysis of RWA.

Peaks	Retention Time	Compound	[M-H]^−^	mg/kg
1	26.12	Cichoric acid	473	259.47 ± 2.57
2	26.81	Caffeic acid	179	189.42 ± 1.08
3	27.70	Apigenin	593	215.18 ± 1.87
4	33.34	Luteolin	593	186.74 ± 2.51
5	34.90	Quercetin	477	173.68 ± 3.05
6	43.58	Catechin	289	189.71 ± 1.93
7	47.69	P-coumaric acid	325	173.28 ± 2.49

**Table 3:** Inhibition diameters of RWA tested against bacterial strains.

Diameter of the Inhibition Zone (mm)					
Strain	RWA	Kanamycin	Oxacillin	Streptomycin	Ceftizoxime
*E. coli*	19.51 ± 0.74	0	11.71 ± 0.54	0	0
*K. pneumoniae*	18.36 ± 1.73	0	0	0	0
*A. baumanii*	16.21 ± 1.45	0	0	0	0
*S. pneumoniae*	17.5 ± 1.08	0	0	0	0
*S. aureus*	15.24 ± 1.31	0	9.89 ± 0.94	0	0

Values are expressed as mean ± SD.

**Table 4:** MIC values of RWA tested against bacterial strains.

Minimum Inhibitory Concentration (mg/mL)					
Strain	RWA	Oxacillin	Streptomycin	Kanamycin	Ceftizoxime
*E. coli*	2.13 ± 0.82	0.041 ± 0.001	0.052 ± 0.003	0.031 ± 0.003	0.064 ± 0.009
*K. pneumoniae*	2.45 ± 0.32	0.041 ± 0.005	0.026 ± 0.007	0.023 ± 0.006	0.023 ± 0.001
*A. baumanii*	3.08 ± 0.27	0.039 ± 0.004	0.024 ± 0.007	0.014 ± 0.007	0.016 ± 0.004
*S. pneumoniae*	2.35 ± 0.64	0.045 ± 0.003	0.032 ± 0.005	0.022 ± 0.003	0.027 ± 0.003
*S. aureus*	4.83 ± 0.76	0.036 ± 0.002	0.029 ± 0.001	0.012 ± 0.004	0.017 ± 0.001

Values are expressed as mean ± SD.

**Table 5:** MICs and inhibition diameters of RWA tested against fungal strains.

	Inhibition Diameter Zone (mm)		Minimum Inhibitory Concentration (mg/mL)	
Strains	RWA	Fluconazole	RWA	Fluconazole
*C. albicans*	29.00 ± 1.51	32.08 ± 1.36	2.04 ± 0.61	3.21 ± 0.04
*A. niger*	28.41 ± 1.08	35.45 ± 1.28	2.84 ± 0.61	2.44 ± 0.08
*A. flavus*	25.65 ± 1.14	31.69 ± 1.92	5.71 ± 0.54	2.52 ± 0.03
*F. oxysporum*	26.71 ± 1.45	37.74 ± 1.34	3.24 ± 0.38	3.68 ± 0.04

Values are expressed as mean ± SD.

## Data Availability

All data related to this work have been presented and its results were disclosed.

## Sample Availability

Samples of RWA are not currently available from the author.
